# Association of the Triglyceride–Glucose Index with Systolic and Diastolic Blood Pressure Among Patients With Type 2 Diabetes Mellitus

**DOI:** 10.7759/cureus.117106

**Published:** 2026-09-28

**Authors:** Mitha K V, Smruthi V, Pibini Paul

**Affiliations:** 1 Physiology, Malabar Medical College Hospital and Research Centre, Calicut, IND

**Keywords:** diastolic blood pressure, insulin resistance., systolic blood pressure, triglyceride glucose index, type 2 diabetic mellitus

## Abstract

Background

Hypertension and type 2 diabetes mellitus (T2DM) frequently coexist, substantially increasing cardiovascular morbidity and mortality. Insulin resistance is a key mechanism linking these conditions. The triglyceride-glucose (TyG) index, derived from fasting triglyceride and glucose levels, has emerged as a simple surrogate marker of insulin resistance. While TyG has been associated with blood pressure in non‑diabetic populations, its relationship with systolic blood pressure (SBP) and diastolic blood pressure (DBP) among patients with established T2DM remains less clearly defined.

Methods

A cross‑sectional study was conducted among 219 patients with T2DM (149 [68.0%] males, 70 [32.0%] females) attending a tertiary care hospital in Kerala, India. Of these, 109 (49.8%) had good glycemic control and 110 (50.2%) had poor glycemic control. Demographic, anthropometric, and biochemical data were collected using standardized procedures. SBP and DBP were measured using a standardized protocol. The TyG index was derived from fasting triglyceride and glucose values. Pearson’s correlation and multiple linear regression analyses (adjusted for age, gender, and BMI) were performed to examine associations between TyG and blood pressure components.

Results

A total of 219 patients with T2DM (149 [68.0%] males, 70 [32.0%] females) were included, of whom 109 (49.8%) had good glycemic control and 110 (50.2%) had poor glycemic control. The mean SBP was 140.0 ± 18.0 mmHg, and the mean DBP was 97.1 ± 11.4 mmHg. The mean TyG index was 10.10 ± 0.44. Pearson’s correlation analysis demonstrated a weak but statistically significant positive association between TyG and DBP (r = 0.149, p = 0.027), while no significant correlation was observed with SBP (r = 0.035, p = 0.612). In regression analysis adjusted for age, gender, and BMI, TyG remained independently associated with DBP (B = 3.52, p = 0.047) but not with SBP.

Conclusion

In patients with T2DM, the TyG index demonstrated a weak but significant association with diastolic blood pressure, whereas no relationship was observed with SBP. These findings suggest that TyG may provide complementary insights into the metabolic determinants of diastolic hypertension. However, given the modest strength of association and low explanatory power, larger prospective studies are required to validate its predictive utility.

## Introduction

Lifestyle diseases are rapidly emerging as a major public health challenge in the modern world, significantly affecting population health and overall well‑being. Among these, hypertension is a critical determinant of cardiovascular health. Often referred to as the “silent killer,” hypertension contributes to sudden cardiac events and premature mortality [1]. Similarly, type 2 diabetes mellitus (T2DM) is another prevalent lifestyle disorder that progressively undermines health, increasing the risk of cardiovascular complications and reducing quality of life [2]. Individuals with diabetes or insulin resistance have a markedly increased risk of developing hypertension. The coexistence of these two conditions substantially elevates cardiovascular morbidity and mortality [3]. Epidemiological evidence indicates that approximately 70% of patients with T2DM are affected by hypertension, underscoring the strong interrelationship between metabolic dysfunction and vascular health [4,5]. This dual burden contributes significantly to the global rise in cardiometabolic disease and highlights the need for simple, reliable markers that can identify patients at risk of elevated blood pressure within diabetic populations.

The triglyceride-glucose index (TyG) has emerged as a promising surrogate marker of insulin resistance [6]. It is calculated from fasting triglyceride and glucose levels, both of which are routinely measured in clinical practice. Elevated fasting glucose is a hallmark of diabetes mellitus, while increased triglyceride levels are closely associated with insulin resistance, metabolic syndrome, and cardiovascular disease risk [7]. Previous studies have demonstrated the predictive role of TyG in relation to diabetes, non‑alcoholic fatty liver disease, and metabolic syndrome. Moreover, evidence from studies in non‑diabetic populations suggests that TyG is significantly and independently associated with elevated blood pressure [8-10].

However, most studies have focused on non-diabetic or general populations, with limited evidence from South Asian cohorts with established T2DM. Therefore, the primary objective of this study was to examine the association of the TyG index with systolic blood pressure (SBP) and diastolic blood pressure (DBP) separately among patients with established T2DM attending a tertiary care teaching hospital in North Kerala.

## Materials and methods

This hospital-based cross-sectional study was conducted in a tertiary care teaching hospital in North Kerala, India, over a seven-month period from June to December 2025. Institutional ethical clearance was obtained prior to commencement (MMCH&RC/IEC/2025/May/01). Written informed consent was obtained from all participants.

Sampling and participants

Consecutive sampling method was employed. All eligible adult patients aged ≥18 years with a confirmed diagnosis of T2DM attending the Department of Medicine during the study period were invited until the required sample size was achieved.

Inclusion criteria

The study included patients aged ≥18 years with confirmed T2DM who provided informed consent.

Exclusion criteria

Patients were excluded if they had type 1 diabetes mellitus, gestational diabetes, age <18 years, unwillingness to consent, and serious comorbid illnesses such as advanced cardiovascular disease, chronic kidney disease, liver failure, or malignancy, as determined by documented medical history, hospital records, ongoing treatment, and physician diagnosis.

Data collection

Demographic data (age, gender) were collected using a structured questionnaire (Appendix). Anthropometric measurements (height, weight) were obtained using standard procedures, and BMI was calculated as weight (kg)/height (m)².

Blood pressure measurement protocol

Blood pressure was measured by trained nursing staff using a calibrated digital sphygmomanometer with appropriate cuff size selected based on mid-arm circumference. Participants were seated with back support and feet flat on the floor, after 5 minutes of rest, with no caffeine, smoking, or exercise in the preceding 30 minutes. Two readings were taken 2 minutes apart in the right arm at heart level, and the average of the two readings was used for analysis. If the difference was >5 mmHg, a third reading was taken, and the average of the last two was considered. Blood pressure was recorded in mmHg.

Biochemical analysis

Each participant was assigned a unique ID to retrieve data from the central laboratory. After overnight fast (8-12 hours), venous samples were collected under aseptic conditions. Fasting plasma glucose was measured using the hexokinase/G-6-PDH method, HbA1c by enzymatic assay, triglycerides using the glycerol phosphate oxidase (GPO) method, and high-density lipoprotein by enzymatic colorimetric assay using an automated chemistry analyzer as per standard operating procedures.

TyG index was calculated using the following formula:

$\mathrm{TyG} = \ln\left(\frac{TG \times FPG}{2}\right)$,

where TG and FPG are in mg/dL.

Operational definitions

Hypertension was defined as SBP ≥140 mmHg and/or DBP ≥90 mmHg or known hypertensive on antihypertensive therapy. Good glycemic control was defined as HbA1c <7.0% and poor control as HbA1c ≥7.0% as per American Diabetes Association guidelines. Comorbidities were defined based on documented medical records and ongoing treatment.

The sample size was calculated using the single population proportion formula:

$n = \frac{(1.96)^2 \times 0.5 \times 0.5}{(0.07)^2}$,

where Z = 1.96 at 95% CI, p = expected prevalence of high TyG (51.5%) from previous literature, q = 48.5, d = allowable error taken as 13% relative error (6.695), and n = (1.96² × 51.5 × 48.5)/6.695² ≈ 214. To ensure adequacy, 219 participants were enrolled. There was no missing data for primary variables; complete case analysis was performed.

Statistical analysis

Analyses were performed using Jamovi version 2.7.17.0. Continuous variables were summarized as mean±SD, median, and min, max. Normality was assessed using the Shapiro-Wilk test. Pearson’s correlation was used for association between SBP, DBP and TyG. Multiple linear regression was conducted separately for SBP and DBP as dependent variables, with TyG, age, gender, and BMI as predictors. Model fit was assessed using R, R², adjusted R², and overall F-test. A p-value of <0.05 was considered significant.

## Results

Study population

A total of 219 participants, 149 (68.0%) males and 70 (32.0%) females, aged 18 years and above, with a confirmed diagnosis of T2DM who consented to participate were included. Among these 219, 109 (49.8%) had good glycemic control, while 110 (50.2%) had poor glycemic control.

Baseline characteristics

The baseline characteristics of the study population are summarized in Table 1. The mean age was 59.2 ± 12.2 years, and the mean BMI was 24.6 ± 3.93 kg/m2. The mean SBP was 140.0 ± 18.0 mmHg, mean DBP was 97.1 ± 11.4 mmHg, mean TyG index was 10.1 ± 0.44, and mean HbA1c was 6.96 ± 0.95%.

**Table 1:** Baseline characteristics of T2DM patients SBP, systolic blood pressure; DBP, diastolic blood pressure; TyG index, triglyceride-glucose index; HbA1C, glycated hemoglobin; BMI, body mass index; T2DM, type 2 diabetes mellitus

Variable	N	Mean ± SD
SBP (mmHg)	219	140.0 ± 18.0
DBP (mmHg)	219	97.1 ± 11.4
TyG index	219	10.1± 0.44
HbA1c (%)	219	6.96 ± 0.95
Age (years)	219	59.2 ± 12.2
BMI (kg/m^2^)	219	24.6 ± 3.93

Correlation analysis

The correlation between TyG index and blood pressure was assessed using Pearson’s correlation analysis (Table 2). A statistically significant weak positive correlation was found between TyG index and DBP (r = 0.149, p = 0.027). No significant correlation was observed between TyG index and SBP (r = 0.035, p = 0.612).

**Table 2:** Pearson's correlation analysis of the TyG index with blood pressure *A p-value of <0.05 was considered statistically significant. SBP, systolic blood pressure; DBP, diastolic blood pressure; TyG index, triglyceride-glucose index

Variable pair	Pearson’s r	p-Value
TyG index - SBP	0.035	0.612
TyG index - DBP	0.149	0.027*
SBP-DBP	0.07	0.301

Regression analysis

Multiple linear regression analysis was conducted to determine predictors of SBP and DBP (Table 3). For SBP, age (B = 0.203, SE = 0.09, p = 0.042) and gender (B = 5.584, SE = 2.692, p = 0.039) were significant predictors. The SBP model was not significant overall (R = 0.194; R² = 0.0375; adjusted R² = 0.0195; F(4,214) = 2.09; p = 0.084). For DBP, the TyG index was a significant predictor (B = 3.52, SE = 1.76, p = 0.047). The DBP model overall was not significant (R = 0.184; R² = 0.0337; adjusted R² = 0.0157; F(4,214) = 1.87; p = 0.117).

**Table 3:** Regression analysis for SBP and DBP SBP model: R = 0.194; R² = 0.0375; adjusted R² = 0.0195; F(4,214) = 2.09; p = 0.084. DBP model: R = 0.184; R² = 0.0337; adjusted R² = 0.0157; F(4,214) = 1.87; p = 0.117. Confidence interval = 95%, B = unstandardized regression coefficient, SE = standard error. *A p-value of <0.05 was considered statistically significant. SBP, systolic blood pressure; DBP, diastolic blood pressure; TyG index, triglyceride-glucose index; BMI, body mass index

Predictor	SBP, B (SE)	p-Value	DBP, B (SE)	p-Value
TyG	0.869 (2.76)	0.753	3.52 (1.76)	0.047*
Age	0.203(0.09)	0.042	0.06 (0.06)	0.365
Gender	5.584 (2.692)	0.039	2.18 (1.72)	0.204
BMI	-0.276 (0.318)	0.386	0.05 (0.20)	0.818

## Discussion

Insulin resistance is considered a central pathophysiological mechanism linking T2DM and hypertension. The TyG index, derived from fasting triglycerides and glucose, has emerged as a simple and reliable surrogate marker of insulin resistance [6]. In the present cohort, the observed blood pressure profile showed a mean systolic blood pressure of 140.0 ± 18.0 mmHg and a mean diastolic blood pressure of 97.1 ± 11.4 mmHg. The mean TyG index was 10.10 ± 0.44, reflecting the underlying metabolic abnormalities.

The key finding of this study was a modest but statistically significant positive correlation between the TyG index and diastolic blood pressure (r = 0.149, p = 0.027), while no significant association was observed with systolic blood pressure. On multivariable regression analysis adjusted for age, gender, and BMI, TyG remained independently associated with DBP (B = 3.52, p = 0.047) but not with SBP. This differential relationship suggests that metabolic disturbances related to insulin resistance may exert a stronger influence on peripheral vascular resistance, which determines diastolic pressure, than on large artery stiffness, which influences systolic pressure. However, vascular resistance and arterial stiffness were not directly assessed in this study, which limits mechanistic interpretation.

Our findings are in line with previous studies in non-diabetic populations reporting positive correlations between TyG and blood pressure [11-13] but extend this evidence to a diabetic cohort for diastolic pressure only. Insulin resistance stimulates sympathetic nervous system activity, increasing catecholamine secretion and peripheral vascular resistance, predominantly affecting DBP [14]. Evidence from type 1 diabetes cohorts showing unchanged central aortic SBP during insulin infusion [15-16] also supports limited sensitivity of SBP to short-term metabolic changes. Thus, DBP may be more responsive to metabolic disturbances, whereas SBP is more determined by structural vascular changes.

Importantly, the observed association was modest, and regression models had low explanatory power (R²<0.04) and overall non-significant F-values. This indicates that TyG and included covariates accounted for only a small proportion of BP variability, highlighting the influence of unmeasured factors such as antihypertensive and lipid-lowering medications, duration of diabetes, lifestyle, and genetic predisposition.

Clinically, TyG may provide complementary information for identifying diabetic patients at risk of diastolic hypertension, which is a recognized risk factor for microvascular complications such as nephropathy and retinopathy. However, given the weak associations and cross‑sectional design, its predictive utility remains limited.

Strengths of this study include a clearly defined T2DM cohort, standardized biochemical assessment using calibrated auto-analyzers, use of the standard TyG formula, and multivariable adjustment for confounders. However, this was a single-center study with consecutive sampling, which limits generalizability, and had a cross-sectional design, which precludes causality. Blood pressure was measured on a single OPD day using Omron HEM-7120 with an appropriate cuff size after 5 minutes of rest with two readings averaged, but ambulatory or repeated day measurements were not available. The association was weak with low R², indicating limited clinical significance, and we did not adjust for antihypertensive, lipid-lowering, and antidiabetic medications, duration of diabetes, diet, physical activity, and smoking. Direct measures of insulin resistance, such as HOMA-IR or clamp and vascular stiffness, were not assessed. Longitudinal multicenter studies with larger samples adjusting for medications and assessing vascular function are needed.

## Conclusions

In this cross-sectional study of 219 T2DM patients, the TyG index showed a weak but significant association with diastolic blood pressure but not with systolic blood pressure. This suggests that TyG may give additional information about metabolic dysfunction and diastolic hypertension in diabetes. However, the association was modest and the model explanatory power was low, and thus results should be interpreted cautiously. Larger prospective studies are needed to confirm whether TyG helps in blood pressure risk stratification and whether lowering TyG improves vascular outcomes.

## Appendices

Questionnaire

 UHID No:

Phone No (WhatsApp):

Section 1: Demographic Data

What is your age? Years

What is your gender? (put tick mark)

Male:

Female:

Other (please specify)            

Section 2: Anthropometric and Clinical Data

Height: cm

Weight: kg
